# The audiological characteristics of infant auditory neuropathy patients without otoacoustic emission

**DOI:** 10.1002/lio2.978

**Published:** 2022-11-24

**Authors:** Kaili Wu, Lan Lan, Wei Shi, Jin Li, Linyi Xie, Fen Xiong, Hongyang Wang, Qiuju Wang

**Affiliations:** ^1^ School of Medical Technology and Information Engineering Zhejiang Chinese Medical University Hangzhou China; ^2^ Department of Audiology and Vestibular Medicine, Senior Department of Otolaryngology, Head and Neck Surgery Chinese PLA General Hospital, Medical School of Chinese PLA Beijing China; ^3^ National Clinical Research Center for Otolaryngologic Diseases Chinese PLA General Hospital Beijing China

**Keywords:** auditory neuropathy, cochlear microphonic, distortion product otoacoustic emission

## Abstract

**Objective:**

To explore the audiological characteristics of infant auditory neuropathy (AN) patients with cochlear microphonic (CM) recorded but absent otoacoustic emission (OAE), clinically reducing the rate of missed diagnosis of AN.

**Methods:**

We retrospectively analyzed the audiological characteristics of infant AN patients in our medical center between 2003 and 2020. A total of 18 infant AN patients were OAE absent group, with CM present and distortion product otoacoustic emission (DPOAE) absent in both ears. A total of 44 infant AN patients were OAE present group, with CM and DPOAE present in both ears.

**Results:**

(1) The found age in OAE absent group was 0.9 (0.02) years old, which was younger than 1.11 (1.63) years old in OAE present group (*p* = .041). (2) The CM threshold of OAE absent group was 80 (10) dB nHL, which was significantly higher (*p* < .001) than OAE present group. CM amplitude were smaller (*p* < .05), and CM duration were shorter (*p* < .05) in OAE absent group. (3) The thresholds of auditory steady‐state response (ASSR) at 0.5, 1, 2, and 4 kHz were 94 (10), 94 (10), 87 (20), and 81 (10) dB HL cg, respectively in OAE absent group, which were higher than those in OAE present group (*p* < .01).

**Conclusions:**

Infant AN patients with CM present and OAE absent showed earlier detection and different audiological performance, which was manifested in ASSR thresholds, audiometric configurations and CM performance. CM thresholds were increased, amplitude and duration were decreased, non‐linearity of I/O function was reduced.

**Level of Evidence:**

4

## Graphical abstract

Explored the characteristics of infant AN patients with CM present and OAE absent, in order to reduce the rate of missed diagnosis of AN. Compared with infant AN patients with both CM and OAE present, the patients with CM present and OAE absent showed earlier onset, worse hearing level, and deterioration of CM performance. Exploring the CM performance changes with the course of the disease may become the direction of future research and further research is needed to explore the influencing factors of CM.

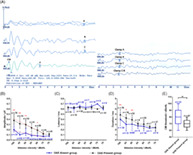

## 1 INTRODUCTION

Auditory neuropathy (AN) is a kind of hearing impairment due to dysfunction of inner hair cells (IHCs), ribbon synapses, spiral ganglion neurons and/or auditory nerve.^1^, ^2^ The main clinical manifestations are that otoacoustic emission (OAE) and/or cochlear microphonic (CM) can be elicited, while auditory brainstem response (ABR) is abnormal or completely absent. Patients could hear sound but could not understand its semantics, and the speech recognition scores decreased disproportionately to the pure tone hearing thresholds.^3^ Several studies have shown that CM is more reliable than OAE in the diagnosis of AN.^4^ The elicitation rate of CM in AN patients was close to 100%, while about 20%–80% of AN patients with OAE present at the first visit, but OAE would disappear during follow‐up (the elicitation rate refers to the proportion of people who meet the elicitation criteria for the test in a population).^5^, ^6^, ^7^

The onset age of AN spans all stages from infants to adolescents and adults. Infant AN refers to the AN with onset or diagnosis in infancy (≤3 years old).^3^ It is difficult for infant AN patients to cooperate with subjective behavior audiometry and speech audiometry. Therefore, OAE and/or CM and ABR are main audiological manifestation of AN in infants. However, OAE in infants is vulnerable to be disturbed by many factors. It is very likely to cause missed diagnosis of AN when OAE absent. For infant AN patients with OAE absent, the performance of other examination results and whether it will be different from the conventional manifestations of typical AN has not been explored. This study focused on the clinical audiological characteristics of infant AN patients with OAE absent, to provide reference for the diagnosis and differential diagnosis of AN.

## 2 MATERIALS AND METHODS

### 2.1 Subjects

The subjects were AN patients admitted to our hospital from 2003 to 2020. The inclusion criteria were as follows: (a) The found age was younger than 3 years old; (b) The diagnosis was bilateral AN; (c) The diagnostic hearing tests performed at our hospital. The exclusion criteria as follows: (a) The diagnosis was unilateral AN; (b) The tympanogram was Type B or C. The found age refers to the age at which parents find that children show hearing loss. The age of testing is the age at which the examination was performed. The course of disease referred to the time interval from the found age to the age of testing.

They were divided into two groups according to whether distortion product OAE (DPOAE) was present. The raw data were examined to measure signal‐to‐noise ratio (SNR) at each f2 frequency in both ears. An SNR >6 dB was considered “present,” otherwise was “absent.” Patients with total tested frequencies absent were included in OAE absent group and with four or more frequencies present in the frequency range of 2–8 kHz were included in OAE present group.^8^

### 2.2 Audiology examination methods

The subjects underwent a comprehensive audiological evaluation, including DPOAE, ABR, CM, auditory steady‐state response (ASSR), 40 Hz—auditory event‐related potential (40 Hz—AERP), behavioral audiometry/pure tone audiometry,^9^, ^10^ and acoustic immittance measurement (see Supplementary S1).

### 2.3 Statistical analysis

Data were coded and entered using the statistical package SPSS version 20.0. For continuous variables, normally distributed data were described using mean and standard deviation, non‐normally distributed data were described using median and quartile, and categorical variables were described using number and percentage. The independent samples *t*‐test, Mann–Whitney test, and χ^2^ or Fisher tests were used to analyze the differences. Correlations between hearing thresholds obtained from different hearing tests were calculated using Pearson correlation analysis. Results were considered to be statistically significant when *p* < .05.

## 3 RESULTS

### 3.1 General characteristics

**FIGURE 1:**
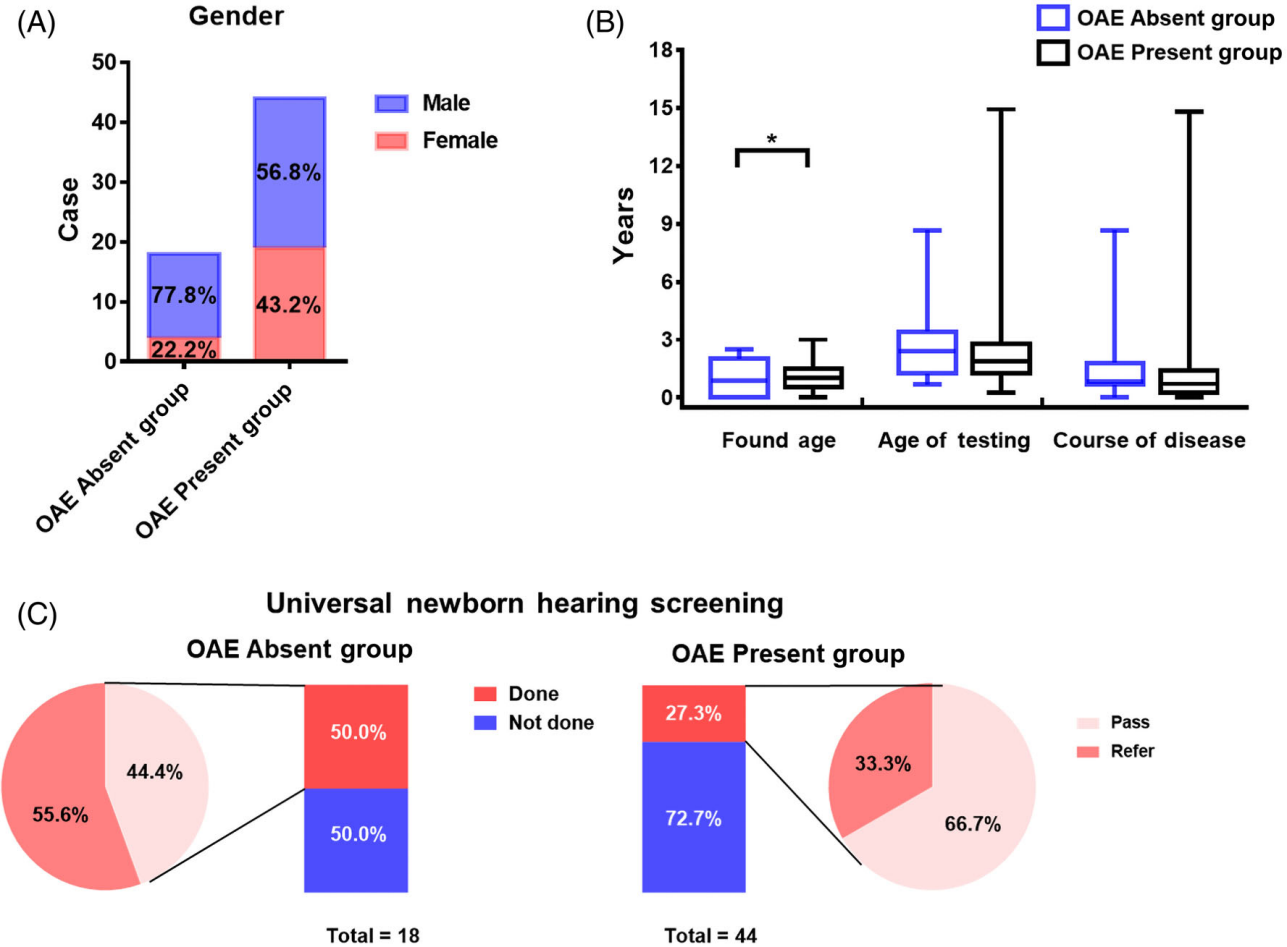
General characteristics of the patients. (A) The gender ratio of the two groups. Blue represented male and red represents female. (B) Comparison of the found age, age of testing, and course of disease between the two group. Blue represented otoacoustic emission (OAE) absent group, and black represented OAE present group. The horizontal line inside the rectangular box represents the median, the upper and lower border lines of the rectangular box represented the upper and lower quartiles, respectively, and the upper and lower short lines outside the rectangular box represented the maximum and minimum values, respectively. (C): Comparison of the composition ratio of universal newborn hearing screening (UNHS). The left picture showed the distribution of the UNHS in OAE absent group, and the right picture showed OAE present group. The bar graph showed the composition ratio of done and not done. Red represented done and blue represented not done. The pie charts showed the composition ratio of the results of UNHS. The color from light to dark represented pass and refer.

A total of 18 patients (36 ears) with no response at all DPOAE frequencies were included in OAE absent group, including 14 males and 4 females. The age of testing was 0.67–8.67 (2.63 ± 1.92) years old. The found age was 0–2.5 (0.90 ± 0.84) years old. The course of disease ranged from 0 to 8.67 (1.74 ± 2.06) years. A total of 44 patients (88 ears) were included in OAE present group, including 25 males and 19 females. The age of testing was 0.25–14.92 (2.37 ± 2.26) years old. The found age was 0–3 (1.10 ± 0.77) years old. The course of disease ranged from 0 to 14.82 (1.26 ± 2.29) years. Less than 50% of the patients underwent universal newborn hearing screening (UNHS). Among the children who underwent UNHS, 57.14% passed the screening (OAE absent vs. present group: 44.44% vs. 66.67%; see Figure 1 and Table S1). All children who have undergone UNHS have at least received OAE screening. All children who passed the screening were only screened for OAE, but not for automatic ABR (AABR).

### 3.2 Audiological characteristics

ABR was mainly absent (OAE absent group: 94.4%; OAE present group: 94.3%), and a few showed only Wave I or V with delayed latency at the maximum stimulus intensity, or the thresholds increased (see Table 1).

**TABLE 1:** Audiological characteristics of patients

Characteristic	OAE absent group (*n* = 36)	OAE present group (*n* = 88)	*p‐*value
Behavioral audiometry/pure tone audiometry			
Grades of hearing loss—no./total no. (%)			
Mild	0	0	.525
Moderate	0	2/36 (5.6)	
Moderately severe	0	3/36 (8.3)	
Severe	2/18 (11.1)	5/36 (13.9)	
Profound	7/18 (38.9)	8/36 (22.2)	
Complete or total	9/18 (50.0)	18/36 (50.0)	
Classifying audiometric configurations—no./total no. (%)			
Flat	7/18 (38.9)	2/36 (5.6)	.013*
Falling	5/18 (27.8)	8/36 (22.2)	
Rising	3/18 (16.7)	10/36 (27.8)	
Peaked or saucer	1/18 (5.6)	8/36 (22.2)	
Trough	0	6/36 (16.7)	
Others	2/18 (11.1)	2/36 (5.6)	
0.5–4 kHz PTA—M (P_25_, P_75_) dB HL	96.25 (81.88, 100.31)	94.38 (75.63, 102.19)	.993
ABR—no./total no. (%)			
Absent	34/36 (94.4)	83/88 (94.3)	.080
Abnormal			
Only wave I at 100 dB nHL	0	1/88 (1.1)	
Wave V latency delay at 100 dB nHL	2/36 (5.6)	0	
The threshold increased significantly	0	4/88 (4.5)	
CM			
Amplitude at 100 dB nHL ‐ M (P_25_, P_75_) μV	0.20 (0.16, 0.28)	0.38 (0.30, 0.50)	<.001**
Latency at 100 dB nHL ‐ M (P_25_, P_75_) ms	0.63 (0.57, 0.65)	0.63 (0.6, 0.65)	.823
Duration at 100 dB nHL ‐ Mean ± SD ms	4.04 ± 1.25	4.88 ± 1.03	.001**
Threshold ‐ dB nHL	80.00 (70.00, 90.00)	70.00 (65.00, 75.00)	<.001**
ASSR threshold—M (P_25_, P_75_) dB HL cg			
500 Hz	94.00 (84.00, 94.00)	74.50 (64.00, 94.00)	<.001**
1000 Hz	94.00 (89.00, 99.00)	89.00 (69.00, 99.00)	.003**
2000 Hz	87.00 (77.00, 97.00)	77.00 (67.00, 87.00)	<.001**
4000 Hz	81.00 (71.00, 81.00)	61.00 (61.00, 71.00)	<.001**
40 Hz‐AERP threshold—M (P_25_, P_75_) dB nHL	100.00 (100.00, 110.00)	100.00 (100.00, 110.00)	.399

Abbreviations: 40 Hz‐AERP, 40 Hz‐auditory event‐related potential; ABR, auditory brainstem response; ASSR, auditory steady‐state response; CM, cochlear microphonic; M (P_25_, P_75_), median (upper quartile, lower quartile); *n*, number of ears; no., number.

* *p* < .05;

** *p* < .01.

**FIGURE 2:**
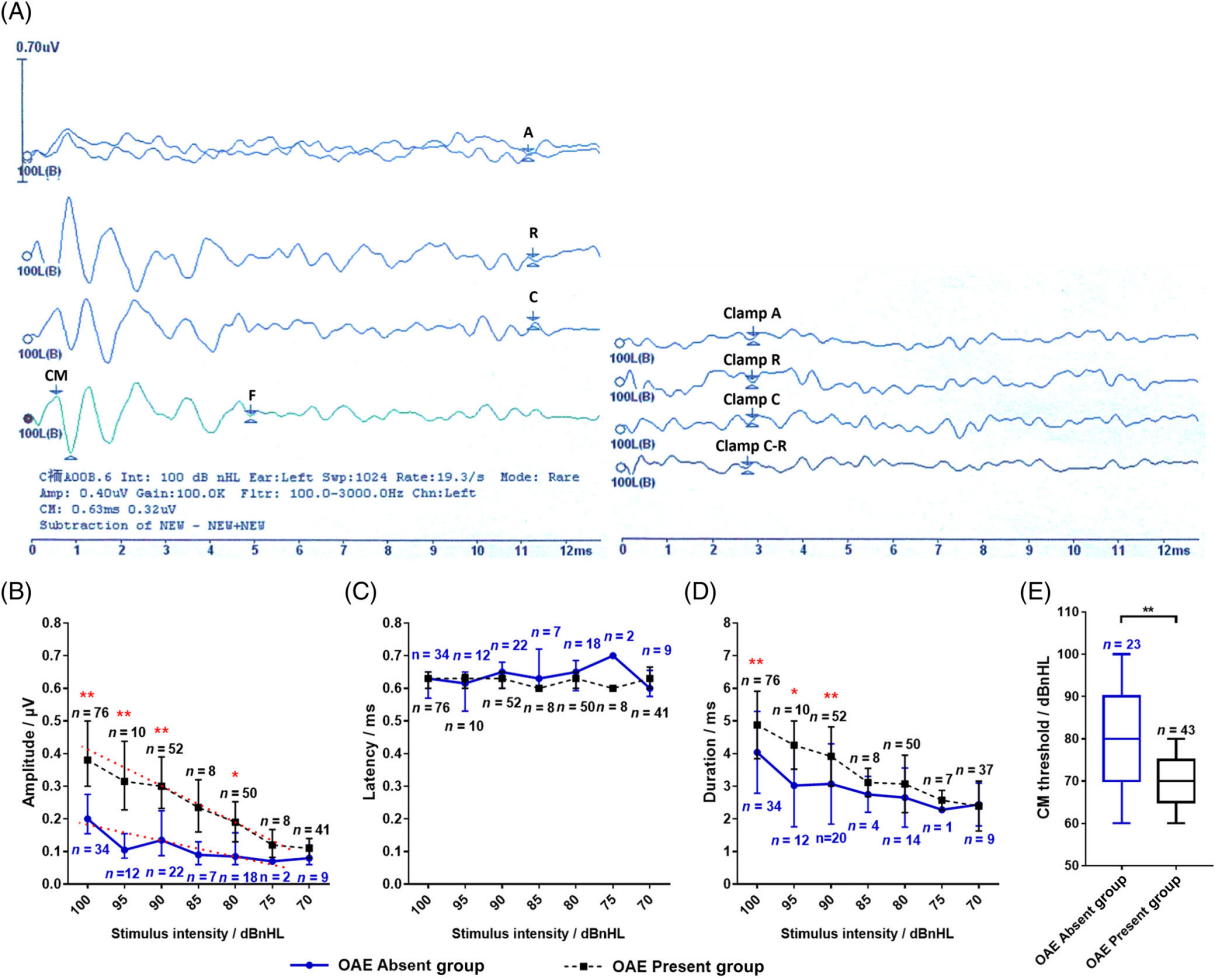
Results of cochlear microphonics (CMs) recorded in subjects (A) An demonstrate of CM waveforms. Each stimulus intensity contains four kinds of waveforms, which was shown on the left. Three of which are generated by alteration (top), rarefaction (second), and condensation (third) clicks, respectively. The other is the CM obtained by subtracting the rarefaction and condensation waves (fourth). The waveforms were obtained repeatedly by sound tube clamped to eliminate the artifact interference, which showed on the right. A represent alteration, C represent condensation, and R represent rarefaction. The superscript of CM is marked at the maximum amplitude within 1 ms, the subscript is marked at the first trough after the peak, and the end time of CM is the moment when the mirror wave disappears. F represent the end time of CM. (B) Comparison of CM amplitudes at different intensities between two groups. (C) Comparison of CM latency at different intensities between two groups. (D) Comparison of CM duration at different intensities between two groups. The blue solid circles represented the median in otoacoustic emission (OAE) absent group, which are connected by blue solid lines; the black solid squares represented the median in OAE present group, which were connected by black dashed lines. The upper and lower short lines represented the upper and lower quartiles, respectively. *n* represents the number of ears, blue represented OAE absent group, and black represented OAE present group. (E) Comparison of CM thresholds between two group. Blue represented OAE absent group, and black represented OAE present group. The horizontal line inside the rectangular box represented the median, the upper and lower border lines of the rectangular box represented the upper and lower quartiles, respectively, and the upper and lower short lines outside the rectangular box represented the maximum and minimum values, respectively. *p < .05; **p < .01.

The CM threshold of OAE absent group was 80.00 (20.00) dB nHL, which was significantly higher than that of 70.00 (10.00) dB nHL in OAE present group (*p* < .001). The amplitude of CM in OAE absent group was 0.20 (0.12) μV lower than that in OAE present group of 0.38 (0.20) μV at 100 dB nHL (*p* < .001). The duration of CM was 4.04 ± 1.25 ms shorter than that in OAE present group of 4.88 ± 1.03 ms at 100 dB nHL (*p* = .001). There was no statistical difference in CM latency between groups at any stimulus intensity. The amplitude and duration of both groups decreased with the decreasing stimulus intensity (see Figure 2 and Table 1).

**FIGURE 3:**
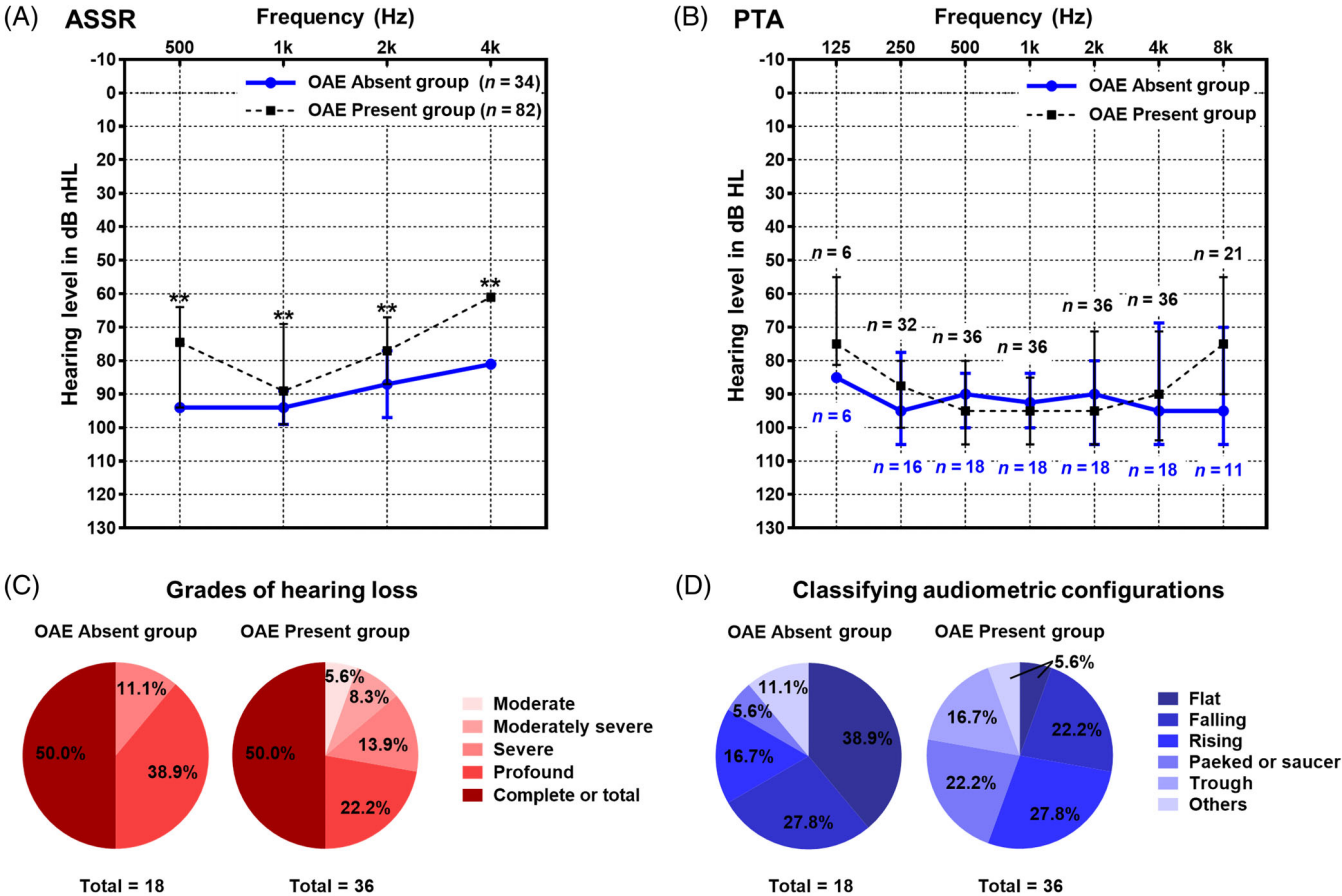
Results of auditory steady‐state response and behavioral audiometry. (A) ASSR hearing thresholds of otoacoustic emission (OAE) absent group and OAE present group. (B) Behavioral audiogram of OAE absent group and OAE present group. The blue solid circles represented the median of each frequency threshold in OAE absent group, which were connected by blue solid lines; the black solid squares represented the median of each frequency threshold in OAE present group, which were connected by black dashed lines. The upper and lower short lines represented the upper and lower quartiles, respectively. n represented the number of ears, blue represented OAE absent group, and black represented OAE present group. (C) Comparison of the grades of hearing loss between the two groups. The left picture showed the distribution of the grade of hearing loss in OAE absent group, and the right picture showed OAE present group. The color from light to dark represented moderate, moderately severe, severe, profound and complete or total deafness respectively. (D) Comparison of the classifying audiometric configurations between the two groups. The left picture showed the distribution of the classifying audiometric configurations in OAE absent group, and the right picture showed OAE present group. The color from dark to light represented flat, falling, rising, peak or saucer, trough, and others, respectively. ASSR, auditory steady‐state response. *p < .05; **p < .01

The ASSR average threshold of OAE absent group was 90.88 (10.00) dB HL cg, which was higher than that of 80.25 (16.25) dB HL cg in OAE present group (*p* = .002). And each frequency threshold of OAE absent group was significantly worse than that of OAE present group (*p* < .05; see Figure 3A and Table 1).

The 40 Hz‐AERP average threshold of the both groups were 100.00 (10.00) dB nHL, and there was no statistical difference between the groups (see Table 1).

The average behavioral hearing threshold in both groups was above 90 dB HL (Figure 3B), which was severe‐profound hearing loss (Figure 3C). In OAE absent group flat type accounted for the largest proportion (38.9%), followed by falling type (27.8%). In OAE present group rising type accounted for the largest proportion (27.8%), followed by falling type (22.2%) and peak or saucer type (22.2%). There was statistical difference in the classifying audiometric configurations between the two groups (*p* = .013; see Figure 3D and Table 1).

**FIGURE 4:**
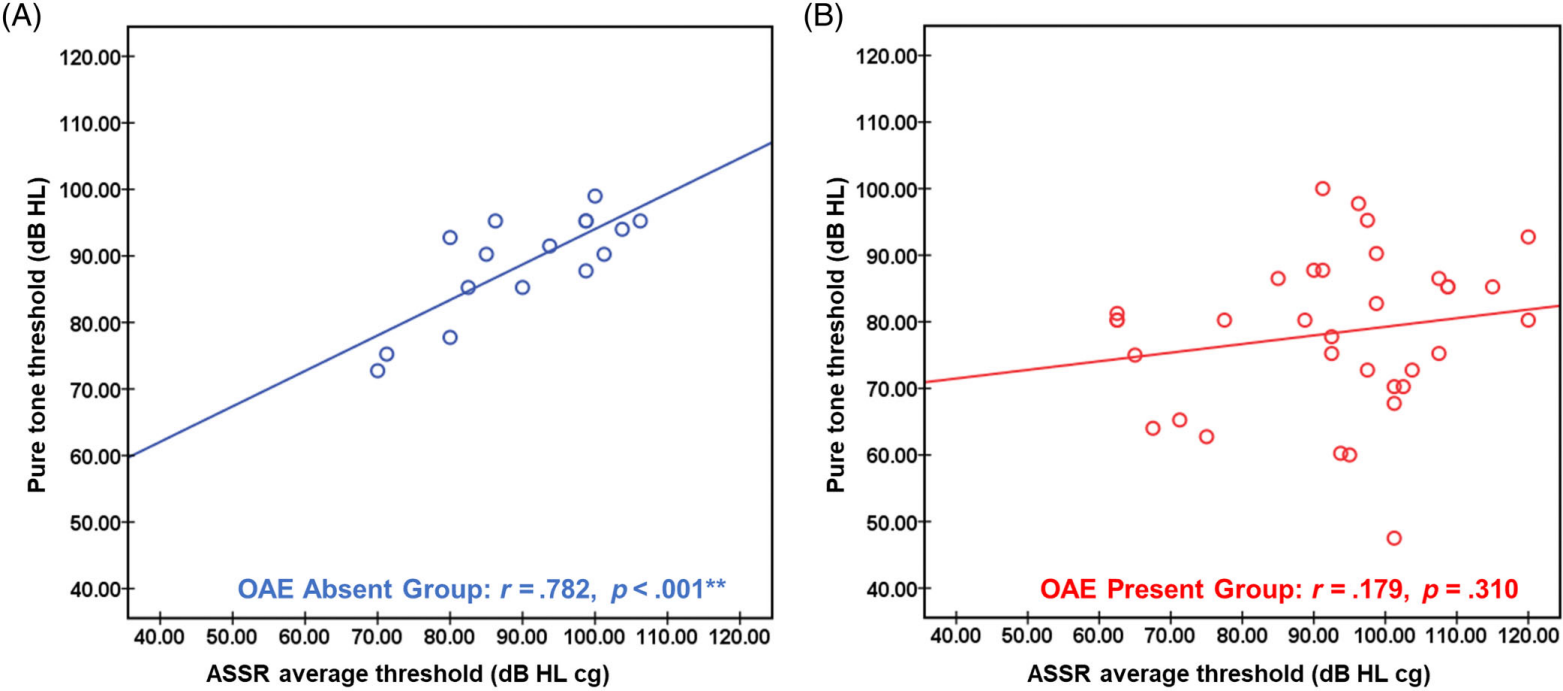
Correlation between behavioral hearing thresholds and auditory steady‐state response thresholds. (A) Correlation between behavioral hearing thresholds and ASSR thresholds in otoacoustic emission (OAE) absent group. (B) Correlation between behavioral hearing thresholds and ASSR thresholds in OAE present group. The open circles represent each case, and the straight lines represent the fitted correlation linear relationship. ASSR, auditory steady‐state response

There was a correlation between the average threshold of behavioral audiometry and the ASSR in OAE absent group (*r* = 0.782, *p* < .001), but no correlation in OAE present group (*r* = 0.179, *p* = .310). Most children showed that the behavioral hearing thresholds were higher than the ASSR thresholds (OAE absent vs. present group: 62.50% vs. 76.47%; see Figure 4).

### 3.3 Follow‐up characteristics

Three cases in OAE absent group were followed up effectively (see Table S1), and the follow‐up periods were 0.5, 1.58, and 3.33 years, respectively. All three patients showed worsened CM performance. In Case 1, under the stimulus intensity of 100 dB nHL, the amplitude of CM decreased, the duration was shortened, the threshold was increased by 5–10 dB in both ears. In Case 2, the CM threshold increased from 70 to 100 dB nHL in the left ear, and the amplitude and duration of CM at 100 dB nHL decreased. The right ear failed to elicit the CM at maximum stimulus intensity. In Case 3, CM could not be recorded at maximum stimulus intensity. Changes in other audiology results were shown in Table S2.

## 4 DISCUSSION

It is variable, heterogeneous and confusing to diagnose infantile AN. Although the protocols and technologies of UNHS have become mature,^11^ some children with AN are still diagnosed in subsequent tests, and cannot be brought to the attention of disease or simply suspected of SNHL through UNHS.

The found age in OAE absent group was significantly younger than OAE present group. They were found at a younger age because they failed the UNHS due to rejection of OAE, while the OAE present group passed the UNHS usually. It suggested that OAE combined with AABR should be performed for UNHS to achieve early detection, diagnosis and intervention of AN.^12^ The elicitation rate of DPOAE was lower than CM in infants and young children.^13^ There was no significant difference in the course of disease and the age of testing between the two groups. Therefore, the disease course may not play a crucial role in DPOAE absence, neither for the age of testing. A total of 4 (44.44%) children passed the screening in OAE absent group, suggesting that OAE could be recorded in the early stage of the disease and disappeared during the progression of the disease.

Both OAE and CM are objective methods to evaluate the function of outer hair cells (OHCs), and CM is more accurate than OAE, especially in infants.^4^ For patients with no DPOAE presence, CM test is suggested since there may be a risk of missed diagnosis of AN. The influence of abnormality of the outer or middle ear, unsuitability of equipment, and improper operation of inspectors^14^, ^15^ on the DPOAE was excluded. DPOAE is more sensitive to hearing loss. If the hearing loss was worse than 40 dB HL, DPOAE might absent.^14^ As AN patients may still have intact OAE despite their severe hearing loss, hearing loss may not be the primary reason of OAE absent. On the contrary, further hearing impairment may be attributable to pathological changes which lead to loss of OAE. As such, the possible mechanism that can explain the reason of OAE absent but CM can be recorded are as follow: (a) OAE is more sensitive to the damage of OHCs.^12^ Previous studies have shown that DPOAE has changed before the change of CM when no obvious structural change is found by electron microscopy.^16^, ^17^ When the OHCs have a certain degree of damage, OAE cannot be elicited but CM can be elicited. CM may be derived from residual OHCs and IHCs, as the function of IHCs may be preserved when the lesion site of AN is synapse or postsynaptic, although its contribution to CM is minimal (15%–20%).^18^, ^19^ (b) OAE and CM may be derived from different aspects of OHCs function. CM was thought to reflect receptor potentials produced at the apical end of OHCs when they were activated mechanically. Receptor potentials played a role in the generation of OAE, but OAE also depended on OHC motility. Conceivably, CM might be recorded in the absence of OAE if receptor potentials remained intact yet the complex mechanisms underlying active processes and OHCs motility in the cochlea were disrupted.^20^

Previous studies showed that the amplitude of CM in AN patients with DPOAE absent was significantly lower than that in normal subjects and AN patients with DPOAE present, and the nonlinear characteristics decreased on the I/O function curve in patients without DPOAE response, with a more linear tendency in amplitude increasing.^15^, ^18^, ^19^, ^20^ In this study, under various stimulation intensities, the CM amplitude of OAE absent group was lower than that of OAE present group, consisted with previous studies. It also found that compared with OAE present group, the CM threshold was higher and duration was shorter in OAE absent group. The reduction of nonlinear characteristics expressed in previous studies could also be obvious in this study. When the stimulus intensity increased from 90 to 100 dB nHL, the amplitude of the OAE absent group increased faster than that of the OAE present group. The nonlinearity of the I/O function curve in the OAE absent group is reduced and closer to linearity. It suggested that the function of IHCs was preserved, and the recorded CM may be derived from IHCs.^18^ The CM is closely related to the stimulus waveform and the vibrational pattern of the basal membrane, including the amplitude and phase of the displacement. CM amplitude directly reflects the stimulus amplitude. The higher the stimulation intensity, the greater the displacement of the basement membrane and the corresponding increase in CM activity.^21^, ^22^

The classifying audiometric configurations of OAE absent group was mostly flat type, while the OAE present group was mostly rising type.^23^ In terms of the grades of pure‐tone hearing loss, which was predominantly severe or profound, and the average threshold was around 95 dB HL in both groups. Previous studies had shown that the phenotype of AN hearing loss ranged from mild hearing loss to total deafness, but it tends to be more severe in AN infants.^12^ OAE present group had lower hearing thresholds at low frequencies and high frequencies (see Figure 3B), indicating that there is more hearing retention, which is different from OAE absent group. The ASSR thresholds of each frequency in OAE absent group were higher than those of OAE present group and were more consistent with the behavioral audiometry results. OAE present group showed the common clinical “√” pattern, with more residual hearing in high‐frequency. Both groups showed that most of the ASSR results were better than the behavioral audiometry results (OAE absent vs. present group: 62.50% vs. 76.47%), which were different from the results of adolescent/adult patients with AN.^3^ It may be due to the severe hearing loss of infants with AN, or related to the children's relatively poor understanding and cooperation. The correlation between ASSR and behavioral audiometry thresholds was still controversial.^24^ In this study, ASSR and behavioral hearing thresholds were just showed moderate correlation in OAE absent group, which seemed to be closer to the characteristics of SNHL.^3^

This article has limitations. First, there were limitations in experimental design and data uniformity as a retrospective study, but the analysis results obtained from existing materials provided direction and reference for subsequent research. For example, the judgment standard of DPOAE results and the influencing factors of CM in patients with AN, and the prognosis of AN patients with OAE absent. Second, follow‐up data were insufficient, only three patients were followed up in OAE absent group. Follow‐up studies for these patients need to be supplemented, and it may be meaningful to observe the changes in their CM. In addition, tracking the prognostic effects and analyzing the prognostic characteristics of such patients, which could be of certain clinical value.

## 5 CONCLUSION

For infant AN patients with CM present and DPOAE absent, the ABR were absent or grossly abnormal, the grades of hearing loss were more than severe, and average hearing threshold was 96.25 dBHL. Compared with AN children with both OAE and CM, the found ages were slightly earlier and audiological performance were different, which was manifested in ASSR thresholds, audiometric configurations and CM performance. CM thresholds were increased, amplitude and duration were decreased, nonlinearity of I/O function was reduced. Except for the CM test, the other hearing tests of infant AN patients with CM present and DPOAE absent might be similar to those of severe‐profound SNHL, so the CM test is of great significance for the diagnosis of these patients. Exploring the changes of CM performance with the course of disease may become the direction of future research.

## CONFLICT OF INTEREST

The authors declare that there is no conflict of interest that could be perceived as prejudicing the impartiality of the research reported.

## Supporting information


**Supplementary S1.** Supplementary methods

**TABLE S1.**General characteristics of the patients.
**TABLE S2.** Follow‐up data of three cases in OAE absent group

## Data Availability

The datasets used and/or analyzed during this study are available from the corresponding author on reasonable request.
