# Supplementary material for: The audiological characteristics of infant auditory neuropathy patients without otoacoustic emission
Source: Laryngoscope Investig Otolaryngol. 2022 Nov 24;7(6):2095–102. doi: 10.1002/lio2.978 (PMC9764789; doi:10.1002/lio2.978)
Supplement: Supplementary file 1 — Supplementary S1. Supplementary methods [file LIO2-7-2095-s002.docx]

**Supplementary Material:**

**Supplementary 1: Methods**

**Audiology Examination Methods**

1. **Evoked Potential Test**

In the electrically shielded sound insulation chamber with background noise lower than 25 dB (A), the auditory brainstem response (ABR), cochlear microphonic (CM), 40 Hz-auditory event related potential (40Hz - AERP), auditory steady-state response (ASSR) and electrocochleogram (ECochG) tests were performed using the IHS smart EP Tester and ER-3A insert earphones.

Subject preparation before the test, including examination of the external ear canal and tympanic membrane, cerumen cleaning, and skin degreasing with 95% alcohol. The recording electrode is placed in the middle of the forehead near the hairline, the reference electrode is placed on the left and right earlobes respectively, the ground electrode is placed at the root of the nose, and the inter - electrode impedance is less than 3 kΩ. For some young patients, oral 10% chloral hydrate is required for sedation and hypnosis before the test.

**ABR Test** ABR was recorded in response to alternating split polarity clicks, of a duration of 10 ms, at a rate of 19.3 pulses per second, the number of sweeps of 1024, a filter setting of 100 - 3000 Hz, over a 12 ms time window, the maximum stimulus intensity of 100 dB nHL,

**CM Test** The recording method is basically the same as ABR test. CMs elicited by rarefaction (R) clicks were subtracted from those elicited by condensation (C) at the same level, and an averaged waveform ((C–R)/2) was obtained. The (C–R)/2 waveform was accepted as the final CM response to be analyzed. To distinguish CMs from artifacts, a patient was retested under same conditions with the plastic tube of the ER-3A earphone clamped.

**40Hz - AERP** 40Hz - AERP was recorded in response to tone burst, of a rise-time of 2 ms, a decay-time of 2 ms, and a duration of 4 ms, the sweep time of 100 ms, the number of sweeps of 512, the start stimulus intensity of 80 dB nHL and the maximum stimulus intensity of 120 dB nHL, During the sweep time, 40 Hz - AERP showed 4 peaks, and the inter-wave latency was about 25 ms, which was judged to be recorded as a response.

**ASSR Test** The stimuli were exponential envelope modulated (AM), the carrier frequencies of 500, 1000, 2000, and 4000 Hz, the modulation frequencies of 78, 85, 93, 101 Hz (left ear), and 79, 87, 95, 103 Hz (right ear), respectively. Filter setting of 30.0 - 300.0Hz, the amplifier gain was 105 times and the artifact rejection was set to 31 μV. Each intensity was set to sweep 400 times, each sweep time was 1.024 s, and the detection frequencies were 500 Hz, 1 kHz, 2 kHz, and 4 kHz.

**ECochG Test** ECochG was recorded in response to alternating split polarity clicks, at a rate of 7.1 pulses per second, the number of sweeps of 500, a filter setting of 100 - 2000 Hz, over a 10 ms time window, the maximum stimulus intensity of 100 dB nHL, The subject lies on his side with the test ear facing up. The reference electrode was placed on the earlobe on the same side of the test ear, the ground electrode was placed at the root of the nose, and a button electrode on the skin surface was used. The recording electrode used the American Sanibel TM tympanic membrane electrode. Check the tympanic membrane markers before testing to rule out abnormalities such as perforation, congestion or thinness. The tympanic membrane was degreased with 95% ethanol cotton wool before placement of the tympanic electrode, and the resistance was less than 50 kΩ. The tip of the tympanic membrane electrode is attached to the posterior lower quadrant of the tympanic membrane surface.

1. **DPOAE Test**

The DPOAE test was performed in the electrically shielded sound insulation chamber with background noise lower than 25 dB (A), using the IHS Smart EP tester, 10D OAE Probe insert earphones. The stimulus is pure tone, frequency ratio of F2 / F1=1.22, stimulus intensity of L1=65 dB SPL and L2=55 dB SPL; A total of 8 frequencies (0.75-8 kHz) are tested. The criterion for judging the effective DPOAE extraction of each frequency is the signal-to-noise ratio ≥ 6 dB.

1. **Behavioral Audiometry \ Pure Tone Audiometry**

Select the appropriate audiometry method according to the subject's age and degree of cooperation. Within 6 months, behavioral observation audiometry (BOA) was used. From 6 months to 2.5 years old, visual reinforcement audiometry (VRA) was used. From 2.5 to 6 years old, play audiometry (PA) was used.

The audiometry was performed in the standard electrically shielded sound insulation chamber, using Madsen Astera2 clinical audiometer and TDH- 39 headphone. The frequency range of the acoustic signal is 0.125 - 8 kHz. The environmental requirements and operation methods comply with the national standard GB/T16403-1996. Record and analyze the hearing threshold of each frequency, the grades of hearing loss, and classifying audiometric configurations.

According to the Hearing Loss Grading Standard issued by the World Health Organization (WHO) in 2021, the mean value of the air conduction hearing thresholds at frequencies of 0.5, 1, 2, and 4 kHz in pure tone audiometry was taken as the average hearing threshold. Hearing loss was graded as follows: normal (< 20 dB HL), mild (20 - 35 dB HL), moderate (35 - 50 dB HL), moderate to severe (50 - 65 dB HL), severe (65 - 80 dB HL), profound (80 - 95 dB HL), complete or total hearing loss / deafness (≥ 95 dB HL) ^1^.

According to the pure tone hearing threshold loss of each frequency, the audiogram can be divided into six types: flat, falling, rising, peak or saucer, trough and notched. Flat: <5 dB rises or fall per octave. Falling: >5 dB increases in threshold per octave. Rising: >5 dB decreases in threshold per octave. Peak or saucer: 20 dB or greater loss at the extreme frequencies, but not at the mid frequencies. Trough: 20 dB or greater loss in the mid frequencies [1,000-2,000 Hz], but not at the extreme frequencies [500 or 4,000 Hz]. Notched: 20 dB or greater loss at one frequency with complete or near-complete recovery at adjacent octave frequencies. Those that cannot be classified as above are other ^2^.

1. **Acoustic Immittance Test**

Use the GSI Tymp Star middle ear analyzer to perform the acoustic immittance examination, including a tympanometry and acoustic reflex threshold. Tympanometry using 226 Hz and 1000 Hz probe tone.

**REFERENCES**

1. WHO. The world report on hearing. Geneva, 2021, 3.
2. Katz J, Chasin M, English K, et al. Handbook of clinical audiology: seventh edition. 2014.
