# Supplementary material for: The audiological characteristics of infant auditory neuropathy patients without otoacoustic emission
Source: Laryngoscope Investig Otolaryngol. 2022 Nov 24;7(6):2095–102. doi: 10.1002/lio2.978 (PMC9764789; doi:10.1002/lio2.978)
Supplement: Supplementary file 2 — TABLE S1.General characteristics of the patients. TABLE S2. Follow‐up data of three cases in OAE absent group [file LIO2-7-2095-s001.docx]

**Supplementary Material:**

**Supplementary 2**

| **Supplementary Table S1. General Characteristics of the Patients.** | | | |
| --- | --- | --- | --- |
| **Characteristic** | **OAE absent Group (N=18)** | **OAE present Group (N=44)** | ***P* Value** |
| Gender - no. (%) |  |  |  |
| Male | 14(77.8) | 25(56.8) | 0.121 |
| Female | 4(22.2) | 19(43.2) |  |
| Age of testing - M (P_25_, P_75_) yr | 3.42(2.36,5.50) | 2.83(1.90,4.83) | 0.232 |
| Found age - M (P_25_, P_75_) yr | 0.90(0.00,0.02) | 1.11(0.26,1.89) | 0.041* |
| Course of disease - M (P_25_, P_75_) yr | 1.53(0.67,4.60) | 1.33(0.58,2.50) | 0.205 |
| Newborns high risk factors - no. (%) |  |  |  |
| Have | 4(23.5) | 12(32.4) | 0.506 |
| Premature birth | 0 | 2 |  |
| Low birth weight | 0 | 1 |  |
| Hyperbilirubinemia | 3 | 11 |  |
| Hypoxia | 2 | 1 |  |
| Virus infection | 1 | 0 |  |
| None | 13(76.5) | 25(67.6) |  |
| Unknow | 1 | 7 |  |
| Universal newborn hearing screening - no. (%) |  |  |  |
| Done | 9(50.0) | 12(27.3) |  |
| Pass | 4 | 8 | 0.396 |
| Refer | 5 | 4 |  |
| Not done | 9(50.0) | 32(72.7) |  |
| Concomitant symptoms - no. (%) |  |  |  |
| Tinnitus | 0 | 1 | 0.328 |
| Limb Dyskinesia | 0 | 2 |  |
| Cerebral palsy | 0 | 1 |  |
| Mental retardation | 0 | 1 |  |
| None | 18 | 39 |  |
| no.: number; N: number of cases; M (P_25_, P_75_): Median (upper quartile, lower quartile); yr: year.  *: *P* < 0.05；**: *P* < 0.01. | | | |

| **Supplementary Table S2. Follow-up data of three cases in OAE absent group** | | | | | | | |
| --- | --- | --- | --- | --- | --- | --- | --- |
| **Characteristic** | | **Case 1** | | **Case 2** | | **Case 3** | |
| Gender | | M | | M | | M | |
| Found age - yr | | 2.00 | | 1.00 | | 1.50 | |
| Newborns high risk factors | | None | | None | | Unknown | |
| Universal Newborn Hearing Screening | | Not done | | Pass | | Pass | |
| Concomitant symptoms | | None | | None | | None | |
| Age of testing - yr | | 3.50 | 4.00 | 1.42 | 3.00 | 2.17 | 5.50 |
| Course of disease - yr | | 1.50 | 2.00 | 0.42 | 2.00 | 0.67 | 4.00 |
| PTA - dB HL | L | 80.00 | 78.75**↓** | NA | 97.50 | NA | 123.75 |
|  | R | 86.25 | 91.25**↑** | NA | 110.00 | NA | 108.75 |
| Classifying audiometric configurations | L | Rising | Rising | NA | Rising | NA | Flat |
|  | R | Rising | Rising | NA | Rising | NA | Flat |
| Tympanogram | L/R | A/A | NA | A/A | A/**C** | A/A | **C**/**C** |
| Acoustic stapedial reflex | | NR | NA | NR | NR | NR | NA |
| DPOAE | L | NR | NR | NR | NR | NR | NR |
|  | R | NR | NR | NR | NR | NR | NR |
| ABR | L | NR | NR | V=6.35ms | NR | NR | NR |
|  | R | NR | NR | V=6.00ms | NR | NR | NR |
| CM (100 dB nHL) | Amplitude (L) -μV | 0.46 | 0.32**↓** | 0.14 | 0.08**↓** | 0.13 | NR**↓** |
|  | Latency (L) - ms | 0.70 | 0.60**↓** | 0.72 | 0.82**↑** | 0.63 | NR**↓** |
|  | Duration (L) - ms | 6.65 | 4.83**↓** | 2.30 | 2.17**↓** | 3.63 | NR**↓** |
|  | Amplitude (R) - μV | 0.38 | 0.27**↓** | 0.29 | NR**↓** | 0.19 | NR**↓** |
|  | Latency (R) - ms | 0.57 | 0.57 - | 0.65 | NR**↓** | 0.60 | NR**↓** |
|  | Duration (R) - ms | 6.53 | 3.17**↓** | 3.42 | NR**↓** | 3.98 | NR**↓** |
| CM threshold - dB nHL | L | 60 | 75**↑** | 70 | 100**↑** | NA(<100) | 100**↑** |
|  | R | 70 | 75**↑** | 75 | NR**↑** | NA(<100) | NR**↑** |
| ASSR - dB nHL | L | 92.75 | NA | 92.75 | NR**↑** | 99.00 | NA |
|  | R | 90.25 | NA | 84.00 | NR**↑** | 85.88 | NA |
| 40Hz-AERP - dB nHL | L | 110 | NA | 120 | NA | 110 | NA |
|  | R | 110 | NA | 120 | NA | 110 | NA |
| yr: years; PTA: pure tone audiometry; DPOAE: distortion product otoacoustic emission; ABR: auditory brainstem response; CM: cochlear microphonic; ASSR: auditory steady-state response; 40Hz-AERP: 40Hz-auditory event related potential.  NA: not available; NR: no reaction. | | | | | | | |
